# Development of High Dietary Fibre-Enriched Cupcake Using Pomegranate Peel Powder

**DOI:** 10.1155/2022/6461949

**Published:** 2022-07-09

**Authors:** Mohamed Gadallah, Zeinab Shabib, Taghreed El-Hazmi

**Affiliations:** ^1^Food Science and Human Nutrition Dept., College of Agriculture and Veterinary Medicine. Qassim University, Buraidah, Saudi Arabia; ^2^Food Science Dept., Faculty of Agriculture, Ain Shams University, Cairo, Egypt; ^3^Food Technology Research Institute, Agriculture Research Center, Giza, Egypt

## Abstract

The effect of substituted wheat flour with pomegranate peel powder (PPP) at different ratios 5, 10, 15, and 20% on chemical composition, physical properties, color appearance, staling, and the sensory evaluation of the high-fibre cupcake was evaluated. The obtained results revealed that a slight increase in ash of cake samples with all levels of PPP was found. The highest value of ash was 1.92% given by cupcakes with 20% PPP. It was observed that a gradual significant (*p* ≤ 0.05) increase in dietary fibre of prepared cupcakes with PPP compared to control cake. The dietary fibre values ranged from 2.73% for the control cake to 17.00% with 20% of PPP; it may be due to the high content of ash and dietary fibre in PPP. Also, using PPP in cupcake manufacturing had a lowered effect on their volume and specific volume, where control cupcake recorded 48.00 cm^3^ and 1.40 cm^3^/g for volume and specific volume, respectively. In addition, a gradual significant (*p* ≤ 0.05) decrease in *L* value (lightness) with incorporated different levels of PPP was found. The control cupcake had the highest lightness value (65.97) with a significant (*p* ≤ 0.05) increase compared to all other cupcake samples that ranged between 37.67 and 30.18 by 5% and 20% of PPP, respectively. The results indicated that the highest value of taste was 18.75, which was given by cupcake with 5% PPP. While increasing the percentage of PPP in cupcake, the taste is less acceptable by panelists, and perhaps, this decreases in taste due to the high ratio of fibre in PPP. The highest staling was given by cupcake replaced with PPP at 20% after the ninth day of storage. Finally, it can be concluded that PPP can be used in cupcake production to raise its dietary fibre and mineral content up to a substitution rate of 10% PPP while retaining acceptable organoleptic properties.

## 1. Introduction

Pomegranate (*Punica granatum* L.) is considered one of the oldest fruits and the earliest to appear in human diet [1]. Nonedible components of pomegranate fruit are considered waste materials like peels and seeds that contain higher amounts of bioactive components compared to the edible part of the fruit [2]. Pomegranate peel residues from juice processing industries were recorded to contain several bioactive compounds, minerals, and dietary fibre for an extended range of human dietary requirements [3]. Human-based studies have indicated that pomegranate has the potential as a protective agent of several diseases, while the food industry's demand for antioxidants and antimicrobials from natural sources, the application of pomegranate, and its extracts (antioxidants and antimicrobials), has been studied extensively in different food products with satisfactory results.

Pomegranate peel powder is a good source for cake making due to its high levels of total polyphenols (1.261%) in addition to its promising source of dietary fibre (12.17%) and organic materials that give health benefits such as prevention of cardiovascular disorders, lowering of blood sugar, anti-inflammation, and antiparasite [4]. Thus, it was recommended that pomegranate peels be commonly used to treat various disorders such as colitis, headache, dysentery, and ulcers [5]. Pomegranate peel contain antioxidant and antimicrobial activities demonstrated by bioactive compounds in their composition. It has successfully seen the use of pomegranate powder and crust extracts in various food products including meat and meat products, edible oils, bakery products, and jelly [6, 7].

Dietary fibre and phytochemicals can be found in fruit and vegetable waste. Fibre derived from fruits and vegetables has a much higher content of soluble dietary fibre, while grain fibre contains cellulose and hemicellulose which are more soluble [8, 9]. Many epidemiological studies confirm that dietary fibre utilization helps prevent or reduce some cancer tumor kinds, obesity, and cardiovascular diseases. Therefore, the fibre consumption recommended is up to 20 to 35 g daily [10].

Cakes are the most utilized bakery product owing to their unique products and are always used in celebrations [11]. This is mainly because they are ready-to-eat products and are available in multiple varieties and at low cost. It is usually made from wheat flour at low extraction (70%) causing deficiency in dietary fibre and phytochemicals. Now, dietary fibre from alternative sources is obtainable which may provide dietary fibre and bioactive compounds as natural components, like vegetable, fruit, and their residues [12].

In order to take advantage of the nutritional and functional properties of fibre, some foods rich in dietary fibre are being developed. There has been an increase in interest in pomegranate as a medicinal and nutritional product because of its multifunctional and great benefit in the diet as it contains several groups of substances that are useful in reducing the risk of disease [9].

Therefore, the study is aimed at improving the nutritional quality of cupcake and studying the impact of incorporating different levels of pomegranate peel powder on chemical, physical, sensory properties and staling rate of prepared high-fibre cupcake.

## 2. Materials and Methods

### 2.1. Materials

Pomegranate (*Punica granatum* L.) peels were obtained from fresh fruits, which were purchased from a local market in Qassim Region, KSA. Wheat flour (70% extraction rate) was obtained from Saudi Grains Organization (SAGO). All other ingredients such as sugar, fresh whole eggs, full-fat milk powder, shortening, baking powder, vanilla, and salt were purchased from the local market in Qassim Region. The chemicals used in the research were obtained from Arkan Development Company Limited at Qassim Region, KSA.

### 2.2. Methods

#### 2.2.1. Preparation of Pomegranate Peel Powder

Pomegranate peel powder (PPP) was prepared according to the method described by Ben-Jeddou et al. [13]. Pomegranate peels were separated from the pomegranate fruits, washed with water to remove any adherent parts of the fruits, and then drained from the water. They were cut into small parts and left to dry in an air-drying oven at 50°C for 24 hours. Then they converted to powder by grinding in a laboratory mill while being sifted using a sieve (0.5 mm). It should be kept in sealed glass containers at ambient temperature (23°C ± 2) until use and analysis.

#### 2.2.2. Determination of Functional Properties

Wheat flour and PPP used in the preparation of cupcakes samples were analyzed for their functional properties which are related to the quality of the prepared cakes. Water holding capacity (WHC) and oil holding capacity (OHC) were measured according to the methods described by Alkarkhi et al. [14]. The specific density was determined using a density bottle at 20°C according to the method given by Kaur and Singh [15]. The swelling index was evaluated according to the method described by Robertson et al. [16].

#### 2.2.3. Preparation of Cake Samples

Cupcake was prepared according to the method described by AACC [17], and the ingredients are found in Table 1. Sugar and shortening were mixed for 3 min at slow speed and then on medium speed for 2 min. Add the eggs and mix for 2 min at medium speed. After that, add milk powder and baking powder, and mix for 4 min. Place 40 g of prepared cake mixture in cake cups, and bake in an electric oven at 180°C for 30 min. After baking, remove the cake samples from the oven, cool for one hour, and then pack in plastic bags until the analysis.

#### 2.2.4. Proximate Chemical Composition

Moisture, ash, lipids, crude protein, and total dietary fibre content of wheat flour, PPP, and different cupcake samples containing different percentages (0, 5, 10, 15 and 20%) of PPP were determined according to the methods described in AOAC [18]. Total nitrogen-free extract (carbohydrates) was calculated by difference.

#### 2.2.5. Determination of Physical Properties of Cupcakes

Weight (g) of cupcake samples was determined after one hour of baking and cooling. The volume (cm^3^) of different prepared cupcakes was dtermined by the method of displacement of rapeseed according to the method mentioned by AACC [17]. Specific volume (cm^3^/g) of cake was calculated by dividing the volume (cm^3^) by their weight (g).

#### 2.2.6. Determination of Color Attributes

The color parameter L^∗^ (100: white; 0: black), a^∗^ (+, red; -, green), and b^∗^ (+, yellow; -, blue) values of wheat flour, PPP, and prepared cupcake samples were determined using a Hunter Lab Color QUEST II Minolta CR-400 (Minolta Camera, Co., Ltd., Osaka, Japan) according to the method described in [19].

#### 2.2.7. Sensory Evaluation of Cupcakes

Partially substituted cupcake samples with deferent levels of PPP were coded with different random numbers and submitted to sensory evaluation by semitrained panelists of food science and human nutrition department staff. The 20 panelists were asked to rate each sensory attribute using the control cake as the basis for evaluation. Cupcake samples were evaluated for appearance (15), crust color (15), crumb color (15), texture (15), taste (20), odor (20), and overall acceptability (100) according to the method described by AACC [17] compared with the control cake sample.

#### 2.2.8. Determination of Cupcake Staling

The staling rate of different prepared cakes was determined after baking within one hour (zero day) and after 3, 6, and 9 days of storage at room temperature (23°C ± 2) by alkaline water retention capacity method (AWRC %) according to the AACC 56-10 method AACC [17].

#### 2.2.9. Statistical Analysis

The statistical analysis was done with three replicates or more of the previous tests, except for the sensory evaluation which were ten replicates. Data were represented as means ± standard errors. Statistical analysis was conducted with SAS program [20] using one way analysis of variance (ANOVA) followed by Duncan's Multiple Range Test with *p* ≤ 0.05 being considered statistically significant.

## 3. Results and Discussion

### 3.1. Functional Properties of Wheat Flour and Pomegranate Peel Powder

Table 2 shows the results of functional properties of wheat flour and PPP. It could be noticed that water holding capacity (WHC), oil holding capacity (OHC), specific density, and swelling index of PPP were higher than wheat flour but without significant (*p* ≥ 0.05) differences, which recorded 3.66, 2.24, 0.75, and 7.68, respectively. The WHC is the ability of a wet material to hold water when exposed to the force of gravity or external pressure by centrifugation [21]. In addition, the water holding capacity test is one of the important parameters influencing the quality and freshness of bakeries.

### 3.2. Chemical Composition and Color of Wheat Flour and Pomegranate Peel Powder

Chemical composition of wheat flour and PPP was determined, and the obtained results are found in Table 3. It could be observed that the ratio of ash (5.32%) of PPP was significantly (*p* ≤ 0.05) higher than that of wheat flour (0.58%). The dietary fibre value of the PPP was 12.12% compared to 0.99% for the wheat flour. On the other hand, PPP was lower in crude protein and carbohydrate (3.45 and 78.17%, respectively), when compared to wheat flour. The results obtained may be consistent with the findings of Mehder [22]. Therefore, pomegranate peel powder is a good source of dietary fibre and ash, which may be used to prepare high-fibre cakes. Also, these results are close to those by Fadavi et al. [23] and Kingsly et al. [24]. As a result, bread contained more fibre and ash with an increased level of pomegranate peels. These results confirmed that pomegranate peels should be used to fortify foodstuffs with fibre and ash [25].

Table 3 shows significant differences for all color values. The *L* value (42.32), which means the degree of white to black color, recorded the lowest value in the PPP compared to wheat flour (87.95), while the redness (a) and yellowness (b) values of PPP were significantly higher than those for wheat flour (-0.34 and 9.31), respectively. The color is affected by many factors including the type of fruit and the degree of its ripeness. But in particular, through the process of drying the peels, they are subject to certain temperatures causing the nonenzymatic brown discoloration that causes the product to darken.

### 3.3. Chemical Composition of Cupcake Samples

It could be observed in Table 4 that addition of PPP to cupcake resulted in a significant increase in moisture content, which ranged from 13.25% to 11.33% for cake with 5% PPP up to 20% PPP, respectively, compared to control cake sample (9.84). These results may be due to the ability of PPP to bind more amount of water (WHO was 3.66 g/g) when preparing the cake, and it can keep the moisture for long time without loss. Slight increase was found in the ash content of all prepared cupcake samples when incorporating PPP at different levels, and the highest value was 1.92% appeared by cupcake with 20% PPP. In addition, it could be noticed clearly significant (*p* ≤ 0.05) increases in dietary fibre of prepared cupcake with PPP compared to control cake (2.73%). The dietary fibre values ranged from 10.46% for cupcake with 5% PPP to 17.00% with 20% of PPP. It may be due to the high content of ash and dietary fibre in PPP. These results were in agreement with Cansu and Isik [26] and Lotfy and Barakat [27]. They mentioned that a significant increase was found in the ash and fibre content of sponge cake compared with the control sample. The same trend in the results for crude protein and carbohydrate was observed when substituting wheat flour with PPP, whereas cupcake containing PPP had significant gradual decrease in protein content from 10.10% by 5% PPP up to 9.30% by 20% PPP addition when compared to control cake (10.60%). In addition, nitrogen-free extract was negatively affected by using PPP in cake manufacture; it was 63.70% for control sample, and then significantly lowers up to 52.47% at 20% of PPP. The reason for these results is the low protein content (3.45%) of PPP compared to wheat flour (11.73%).

### 3.4. Physical Properties of Cupcake

The results found in Table 5 showed that with increasing levels of PPP in cupcake, weight was gradually increased, while volume and specific volume were decreased significantly when compared to control cake. The highest weight (36.10 g) was given by adding 20% PPP to the cupcake sample with significantly increase compared to 34.20 g for control cake. This is due to high content of PPP in the cake samples caused the cohesion and convergence of molecules, which leads to an increase in weight. Incorporating PPP in cupcake had negative effect on volume and specific volume, where the highest values of 48.00 cm^3^ and 1.40 cm^3^/g were recorded by control cake. These results were in agreement with those obtained by Mehder [22], Lotfy and Barakat [27]. The reason for these results is the gluten of the wheat flour, which traps carbon dioxide and contributes to the rise in volume and the texture becomes spongy, while PPP caused dilution for the gluten level in the cake mixture. Gas retention in dough during baking is a property of wheat flour gluten which becomes strong and extensive. This prevents escape of the gas during baking and allows the dough to rise [28].

### 3.5. Color Parameters of Cupcake

**Figure 1:**
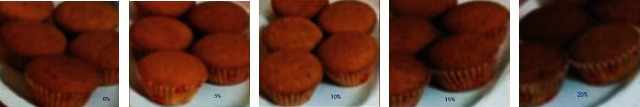
The surface color of prepared cupcake samples containing of PPP.

The results of cupcake sample color replaced with PPP at 5, 10, 15, and 20% are tabulated in Table 6 and shown in Figure 1. It could be observed that a gradual significant decrease in the *L* value (lightness) with incorporated different levels of PPP. The control cake had the highest *L* value (65.97) with significant (*p* ≤ 0.05) increases compared to other cake samples, which ranged between 37.67 and 30.18 by 5% and 20% of PPP respectively. Substituting wheat flour in cake making with PPP at 5, 10, 15, and 20% resulted in clear significant high in redness (a) (5.03, 5.34, 7.70, and 6.37, respectively), when compared to control cake (0.64). These results were attributed to the red color of PPP, which was confirmed with their value 9.28 in Table 3. The control sample recorded the highest *b* value of 25.91, while PPP caused a significant decrease in the *b* value. Cupcakes containing 20% of PPP recorded the lowest significant value at 12.93; on the other hand, no significant differences were observed between cupcake samples containing 5, 10, and 15% of PPP. These results were in agreement with Cansu and Isik [26] who mentioned that cake crumb and crust *L* and *b* values decreased by increasing the amount of pomegranate peel.

The color, whether it is the raw materials used in the manufacture of the product or the color of the product, is considered one of the important factors in the consumer acceptance of the product, because of its direct impact on the components included in the manufacture of the product and the percentage of additives in mixtures and production conditions.

### 3.6. Sensory Evaluation of Cupcake

The results of the sensory evaluation of the partially replaced cupcake with PPP in Table 7 showed that there were no significant differences in appearance values between both the control sample and the cupcake replaced with 5% and 10% PPP. Concerning crust color, it was noted that the cupcake replaced with 20% PPP recorded the lowest value for the crust color, where it was recorded 13.90 with no significant difference between it and other samples replaced with the PPP. There was no significant difference in crumb color of cake between all samples. This is consistent with Ismail et al. [29] as they mentioned that the color of cookies replaced with PPP had the significant impact on sensory evaluation. Similar results were also recorded by Turksoy and Ozkaya [30].

Regarding the textures, cupcake sample with 5% of PPP recorded the highest value of 14.40, whereas the sample with 20% of PPP had the lowest value of 12.90. These results were consistent with the results of Uysal et al. [31] where they indicated that there is a direct correlation between the stiffness of the textures and the percentage of fibre contents added to the processed cake product. Cansu and Isik [26] found that increasing the pomegranate peel level in cakes caused an increase in hardness values of texture and decreasing in crumb cell structure and chewiness scores in sensory analysis. So we suggested increasing the liquid phase ratio in pomegranate powder cake batters.

As for the taste, it could be found that the highest value of taste was recorded by cupcake sample prepared by replacing wheat flour by 5% of PPP (18.75), while the higher the level of PPP, the taste is less acceptable, and perhaps, this decreases in the taste due to the high percentage of PPP fibre. These results were in agreement with those of Bom et al. [32] and Karanewsky et al. [33]. In overall acceptability of the cupcake samples as the sum of the sensory qualities, it could be concluded that wheat flour could be replaced by 5 and 10% of PPP without negative effect on the sensory properties of prepared cupcakes.

### 3.7. Staling Rate of Cupcake

**Figure 2:**
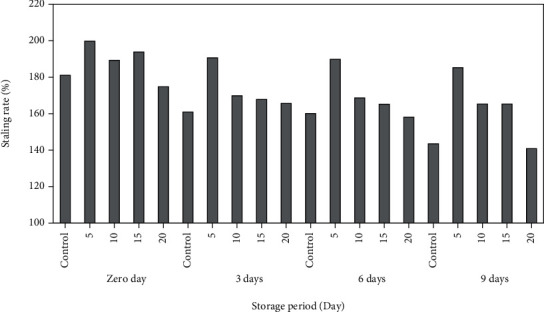
Staling rate (%) for cupcake samples incorporated with PPP. 5: cake containing 5% of PPP, 10: cake containing 10% of PPP, 15: cake containing 15% of PPP, 20: cake containing 20% of PPP.

The effect of storage for 0, 3, 6, and 9 days at room temperature on the staling rate of cupcake samples with PPP was measured. The results illustrated by Figure 2 showed that the ratio of staling for cake samples with PPP during the storage period ranged between 194.66% and 121.00%. The highest freshness (194.66%) was recorded by cupcake sample with 5% PPP ate zero time, because the storage period was very short, and the percentage of staling was low but there are other materials that delay the staling, while the highest level of staling (121.00%) was given by the cupcake sample replaced with PPP at 20% after the ninth day of storage. The high percentage of staling is always associated with increasing the storage period, the more days of storage, and the higher percentage of staling. The results showed a decrease in the percentage of freshness in the cupcake samples containing pomegranate peels at 10, 15, and 20%, and these results are consistent with the results of Mehder [22].

## 4. Conclusion

It could be concluded that the pomegranate peel powder has a functional property higher than those of wheat flour. Also, a high percentage of fibre (17.00%) was recorded for cake with PPP at 20% significant increase in the fibre content compared to control cake (2.73%). With increasing the ratio of PPP to all cupcake samples at different proportions, some physical properties of the cupcake such as volume and specific volume were decreased, while others such as weight and specific weight were increased compared to the control cupcake. In the higher storage period, the higher staling of cupcake samples appeared, and thus, the moisture content of the fortified cupcake decreases with different proportions of PPP. Finally, high-fibre cupcake can be prepared with substituted wheat flour up to 10% with pomegranate peel powder without any negative effects on the quality properties and sensory acceptable of the product.

## Figures and Tables

**Table 1:** Weight (g) of cupcake ingredients incorporated with PPP.

Cake samples	Cake ingredients (g)						
	Wheat flour	PPP	Sugar	Shortening	Fresh egg	Milk powder	Baking powder
Control	100	0	60	50	85	3	4
5% PPP	95	5	60	50	85	3	4
10% PPP	90	10	60	50	85	3	4
15% PPP	85	15	60	50	85	3	4
20% PPP	80	20	60	50	85	3	4

**Table 2:** Functional properties of wheat flour and pomegranate peel powder.

Materials	Functional properties			
	WHC (g/g)	OHC (g/g)	Specific density (g/cm^3^)	Swelling index (cm^3^/g)
Wheat flour	3.15 ± 0.34^a^	1.79 ± 0.03^a^	0.64 ± 0.05^a^	6.83 ± 0.66^a^
PPP	3.66 ± 0.68^a^	2.24 ± 0.32^a^	0.75 ± 0.01^a^	7.68 ± 0.09^a^

PPP: pomegranate peel powder. Data are the mean ± SE; *n* = 3. Values followed by the same letters in the same column are not significantly different (*p* ≤ 0.05).

**Table 3:** Chemical composition (% on dry weight basis) and color of wheat flour and PPP.

Parameters	Wheat flour	PPP
	Chemical composition	
Moisture	11.44 ± 0.29^a^	9.22 ± 0.16^b^
Ash	0.58 ± 0.07^b^	5.32 ± 1.35^a^
Lipids	1.49 ± 0.28^a^	0.92 ± 0.27^a^
Crude protein	11.73 ± 0.30^a^	3.45 ± 0.38^b^
Dietary fibre	0.99 ± 0.28^b^	12.12 ± 0.20^a^
Nitrogen-free extract (NFE)	85.18 ± 0.07^a^	78.17 ± 2.03^b^
	Color values	
L∗	87.95 ± 0.50^a^	42.32 ± 0.15^b^
a∗	−0.34 ± 0.14^b^	9.28 ± 0.02^a^
b∗	9.31 ± 0.05^b^	18.53 ± 0.07^a^

PPP: pomegranate peel powder. Data are the mean ± SE; *n* = 3. Values followed by the same letters in the same column are not significantly different (*p* ≤ 0.05). NFE: calculated by difference. L∗: lightness; a∗: redness; b∗: yellowness.

**Table 4:** Chemical composition (% on dry weight basis) of cupcake containing PPP.

Cake samples	Moisture	Ash	Lipids	Crude protein^∗^	Dietary fibre	NFE^∗∗^ (carbohydrate)
Control	9.84 ± 0.19^d^	1.76 ± 0.11^c^	21.20 ± 0.07^a^	10.60 ± 0.20^a^	2.73 ± 0.29^d^	63.70 ± 0.23^a^
5% PPP	13.25 ± 0.17^a^	1.80 ± 0.02^b^	20.10 ± 0.08^ab^	10.10 ± 0.05^b^	10.46 ± 0.48^c^	57.52 ± 0.50^b^
10% PPP	12.39 ± 0.08^b^	1.81 ± 0.04^b^	20.10 ± 0.10^ab^	9.70 ± 0.08^c^	11.00 ± 0.46^c^	57.38 ± 0.43^b^
15% PPP	12.34 ± 0.04^b^	1.83 ± 0.08^ab^	19.50 ± 0.33^b^	9.60 ± 0.09^cd^	14.73 ± 3.06^b^	54.32 ± 3.40^c^
20% PPP	11.33 ± 0.13^c^	1.92 ± 0.02^a^	19.30 ± 0.08^b^	9.30 ± 0.04^d^	17.00 ± 2.00^a^	52.47 ± 2.11^c^

PP: pomegranate peel powder. Data are the mean ± SE; *n* = 3. Values followed by the same letters in the same column are not significantly different (*p* ≤ 0.05). ^∗^Wheat flour (*N* × 5.70); cake samples (*N* × 6.25). NFE^∗∗^: calculated by difference.

**Table 5:** Physical properties of cupcake samples containing different ratios of PPP.

Cake samples	Weight (g)	Volume (cm^3^)	Specific volume (cm^3^/g)
Control cupcake	34.20 ± 0.20^c^	48.00 ± 0.70^a^	1.40 ± 0.01^a^
Cupcake with 5% PPP	35.00 ± 0.00^b^	42.80 ± 0.48^b^	1.22 ± 0.01^ab^
Cupcake with 10% PPP	35.30 ± 0.20^ab^	37.80 ± 0.37^c^	1.07 ± 0.01^b^
Cupcake with 15% PPP	35.80 ± 0.37^a^	35.40 ± 0.40^d^	0.98 ± 0.00^c^
Cupcake with 20% PPP	36.10 ± 0.20^a^	33.00 ± 0.44^e^	0.91 ± 0.01^c^

PPP: pomegranate peel powder. Data are the mean ± SE; *n* = 3. Mean values in the same column with the same superscript do not differ significantly (*p* ≤ 0.05).

**Table 6:** Color parameters of cupcake with different levels of PPP.

Cake samples	L∗	a∗	b∗
Control cupcake	65.97 ± 0.41^a^	0.64 ± 0.20^c^	25.91 ± 0.09^a^
Cupcake with 5% PPP	37.67 ± 0.70^b^	5.03 ± 0.03^b^	14.64 ± 0.17^b^
Cupcake with 10% PPP	35.07 ± 0.46^c^	5.34 ± 0.03^ab^	14.44 ± 0.15^b^
Cupcake with 15% PPP	33.93 ± 0.24^c^	5.70 ± 0.10^a^	14.21 ± 0.28^b^
Cupcake with 20% PPP	30.18 ± 0.57^d^	6.37 ± 0.16^a^	12.93 ± 0.32^c^

PPP: pomegranate peel powder. Data are the mean ± SE; *n* = 3. Mean values in the same column bearing the same superscript do not differ significantly (*p* ≤ 0.05). L∗: lightness; a∗: redness; b∗: yellowness.

**Table 7:** Sensory evaluation of cupcake samples partially substituted with PPP.

Cake samples	Appearance (15)	Crust color (15)	Crumb color (15)	Texture (15)	Taste (20)	Odor (20)	Overall acceptability (100)
Control	14.50 ± 0.16^a^	14.70 ± 0.15^a^	14.80 ± 0.13^a^	14.20 ± 0.20^ab^	18.30 ± 0.59^a^	19.60 ± 0.16^a^	96.10 ± 0.78^a^
5% PPP	14.70 ± 0.15^a^	14.60 ± 0.16^ab^	14.60 ± 0.22^ab^	14.40 ± 0.30^a^	18.75 ± 0.30^a^	18.95 ± 0.24^ab^	95.90 ± 0.78^a^
10% PPP	14.10 ± 0.23^a^	14.50 ± 0.16^ab^	14.50 ± 0.26^ab^	13.60 ± 0.33^ab^	18.10 ± 0.43^a^	18.10 ± 0.72^b^	93.00 ± 1.29^ab^
15% PPP	13.10 ± 0.43^b^	14.00 ± 0.33^ab^	14.10 ± 0.31^ab^	13.20 ± 0.29^ab^	17.90 ± 0.27^b^	17.90 ± 0.54^b^	89.55 ± 1.97^b^
20% PPP	12.60 ± 0.52^b^	13.90 ± 0.31^b^	13.80 ± 0.32^b^	12.90 ± 0.50^c^	17.25 ± 0.79^b^	15.70 ± 0.33^c^	89.80 ± 1.54^b^

PPP: pomegranate peel powder. Data are the mean ± SE; *n* = 10. Mean values in the same column bearing the same superscript do not differ significantly (*p* ≤ 0.05).

## Data Availability

Data are available at the Food Science and Human Nutrition Dept., College of Agriculture and Veterinary Medicine, Qassim University, Buraidah, Saudi Arabia.
